# Suboptimal care of peanut allergic children in Germany concerning professional dietary advice and the supply of self-injectable epinephrine

**DOI:** 10.1186/2045-7022-3-S3-P38

**Published:** 2013-07-25

**Authors:** V Trendelenburg, K Blümchen, F Ahrens, A Gruebl, E Hamelmann, G Hansen, A Heinzmann, K Nemat, B Niggemann, K Beyer

**Affiliations:** 1 Dept. of Pediatric Pneumology and Immunology, Charité University Medicine, Berlin, Germany; 2 Children’s Hospital “Altona”, Hamburg, Germany; 3 Dept. of Pediatrics, Technical University Munich, Munich, Germany; 4 Dept. of Pediatrics, Ruhr-University Bochum, Bochum, Germany; 5 Dept. of Pediatric Pneumology, Allergology and Neonatology, Hannover Medical School, Hannover, Germany; 6 Center for Pediatrics and Adolescent Medicine, University of Freiburg, Freiburg, Germany; 7 Dept. of Pediatrics, University Hospital Carl Gustav Carus, Technical University of Dresden, Dresden, Germany; 8 Pediatric Allergology and Pneumology, German Red Cross Hospital Westend, Berlin, Germany

## Background

Patients with peanut allergy are advised to strictly avoid peanuts in their diet and to carry an emergency kit including self-injectable epinephrine at all times.

The aim of the study was to determine to which extent peanut allergic children in Germany are provided with self-injectable epinephrine and if they had received professional dietary advice.

## Methods

63 peanut allergic children (median: age 6 years; peanut-specific IgE 73,5kU/l) who participated in an oral immunotherapy (OIT)-study were enrolled. Data concerning age of diagnosis, provision with self-injectable epinephrine, professional advice and performance of a peanut-free diet as well as the characterization of accidental allergic reactions to peanut (grade of severity I-V) were collected at their baseline visit of the OIT-study.

## Results

At baseline visit, 17/63 children (27%) had not been provided with self-injectable epinephrine although the diagnosis of their peanut allergy was already made 4 years (median) ago. Five of these 17 children had at least one severe allergic reaction after accidental peanut consumption (grade IV) in their history. 27/63 children (43%) had not received self-injectable epinephrine at initial diagnosis but later in their life. In 13 of these 27 cases self-injectable epinephrine was prescribed only after at least one severe allergic reaction to peanut (grade IV). 19/63 children (30%) had received self-injectable epinephrine directly at initial diagnosis. A strict avoidance of peanut was performed by 51/63 families (82%). 12/63 families (18%) did not avoid products labelled with “may contain traces of peanuts”. Only 13/63 families (21%) had received dietary advice from a dietician.

## Conclusion

Currently**,** peanut allergic children are not sufficiently provided with self-injectable epinephrine and do not receive enough professional dietary advice in Germany. Accidental allergic reactions are common. Therefore we recommend prescribing an emergency kit including self-injectable epinephrine directly at initial diagnosis. Furthermore families should receive an emergency training and a professional dietary advice.

## Disclosure of interest

None declared.

